# Efficient Traffic Video Dehazing Using Adaptive Dark Channel Prior and Spatial–Temporal Correlations

**DOI:** 10.3390/s19071593

**Published:** 2019-04-02

**Authors:** Tianyang Dong, Guoqing Zhao, Jiamin Wu, Yang Ye, Ying Shen

**Affiliations:** College of Computer Science and Technology, Zhejiang University of Technology, Hangzhou 310023, China; dty@zjut.edu.cn (T.D.); m15695719625@163.com (G.Z.); hzwujiamin@163.com (J.W.); yeyang80@zjut.edu.cn (Y.Y.)

**Keywords:** image dehazing, traffic video dehazing, dark channel prior, spatial-temporal correlation, contrast enhancement

## Abstract

In order to restore traffic videos with different degrees of haziness in a real-time and adaptive manner, this paper presents an efficient traffic video dehazing method using adaptive dark channel prior and spatial-temporal correlations. This method uses a haziness flag to measure the degree of haziness in images based on dark channel prior. Then, it gets the adaptive initial transmission value by establishing the relationship between the image contrast and haziness flag. In addition, this method takes advantage of the spatial and temporal correlations among traffic videos to speed up the dehazing process and optimize the block structure of restored videos. Extensive experimental results show that the proposed method has superior haze removing and color balancing capabilities for the images with different degrees of haze, and it can restore the degraded videos in real time. Our method can restore the video with a resolution of 720 × 592 at about 57 frames per second, nearly four times faster than dark-channel-prior-based method and one time faster than image-contrast-enhanced method.

## 1. Introduction

Today, traffic video analysis plays a very important role in intelligent transportation systems. It has become a common way to help people track a vehicle, as well as locate and judge an accident. Because the images captured by outdoor cameras are often affected by different weather conditions, they suffer from poor visibility and lack of contrast. In the literature, there are many enhancements and dehazing algorithms that improve different images, such as traffic videos, underwater images, and satellite imagery [1,2,3]. The hazy weather that happens frequently all over the world is becoming a video analysis killer. The haze captured in the video degrades the contrast and color information and reduces the visibility. Therefore, the problem of how to efficiently and effectively remove the haze in traffic videos has attracted broad attention from both academia and industry. In general, when dealing with haze removal in traffic videos, the existing dehazing algorithms often exhibit poor real-time performance, overstretched contrast, and even fail to remove dense haze. The key issue of these problems is how to deal with images in different scenes with different degrees of haze, thus an adaptive algorithm that can remove haze based on the image characteristics is needed. Moreover, the existing video-dehazing methods are almost universal for all videos and do not consider the characteristics of videos in particular scenarios. For traffic videos, the time continuity, lane space structure, and camera spatial locations can be effectively used to decrease computational cost.

In order to restore traffic videos with different degrees of haziness in a real-time and adaptive manner, this paper presents an efficient traffic video dehazing method using adaptive dark channel prior and spatial-temporal correlations. This method can avoid overstretched contrast after haze removal and obtain satisfactory restored results for dense hazy videos by using a novel approach involving adaptive transmission estimation. This method also takes full advantage of the temporal and spatial correlations in traffic videos to meet the requirements of real-time dehazing, such as using time continuity to set the time slice, refining transmission by characteristics of block structure, decreasing restored area according to the lane space, and simplifying the calculation of parameters by using multi-camera distribution.

## 2. Related Works

Essentially, videos are composed of frames, thus the haze removal method for images can be used for videos. The image dehazing method is the most common way to restore hazy images. This method considers the inverse process of image degradation and describes the image degradation process in detail through an established physical model. The most critical step of this method is to obtain the parameters of the degradation model. Oakley et al. [4] improved the image quality by using the physical model and estimated the degradation model parameters based on a statistical model. This method is not widely used because it is only useful for gray-scale images, and the acquiring parameters require calibrated radar to get depth information. Narasimhan et al. [5] proposed a method to estimate the depth information by comparing two images of the same scene in different weather conditions. Chen et al. [6] used a sunny image and a foggy image for reference images to calculate parameters. Both of these methods need to receive eligible images in advance, which increases the difficulty of image acquisition.

To obtain the parameters of the degradation model effectively, some dehazing methods based on prior knowledge or assumptions were proposed, and they do not need to get reference images in advance or use an additional hardware device. Therefore, these methods have better adaptability than previous methods. Based on the assumption that a haze-free image has a higher contrast than a hazy image, Tan [7] proposed a haze removal approach by maximizing the contrast of recovered scene radiance. This approach can produce a satisfactory result for haze removal in single images, but it tends to overcompensate for the reduced contrast and leads to halo effects. Fattal [8] decomposed scene radiance of an image into the albedo and shading and then estimated the scene radiance based on independent component analysis, assuming that transmission shading and surface shading are locally uncorrelated. However, this method cannot generate impressive results when the captured image is heavily obscured by fog. He et al. [9] presented a single image haze removal method by using dark channel prior, which can estimate the transmission map directly. However, when a large white area without shading exists in the images, or the images have uneven illumination, this method takes a long time to restore the hazy images. In addition, the use of the soft matting algorithm makes this a complex computation. Then, Lai et al. [10] presented a haze removal method based on the difference-structure-preservation prior. In this method, the difference-structure-preservation dictionary is learned such that the local consistency features of the transmission map can be well preserved after coefficient shrinkage. Zhu et al. [11] presented a simple but effective Color Attenuation Prior (CAP)algorithm similar to Dark Channel Prior (DCP)using the difference in brightness and saturation to estimate the haze concentration to build a depth model for dehazing. Up until now, other researchers have improved their dehazing algorithms based on the dark channel prior. Yeh et al. [12] introduced a haze removal algorithm based on region decomposition and feature fusion, which is especially suitable for hazy images with large sky regions. Li et al. [13] proposed a novel haze removal method based on sky segmentation and dark channel prior to restore images. In this method, the average image intensity of the sky region is chosen as the atmospheric light value. Wang et al. [14] designed a new method of selecting atmospheric light values to weaken the area where the dark channel priority does not work effectively. A visibility restoration method was introduced by Huang et al. [15], which consists of three modules: (i) depth estimation module based on dark channel priority, (ii) color analysis module that repairs depth estimation distortion, and (iii) visibility restoration module that generates repair results. Riaz et al. [16] proposed a new and efficient method for transmission estimation with bright-object handling capability, which uses a local average haziness value to compute the transmission of such surfaces based on the observation that the transmission of a surface is loosely connected to its neighbors.

Usually, traffic video dehazing algorithms are proposed based on single-image dehazing algorithms. However, the computational complexity makes it difficult to apply single-image dehazing algorithms directly to video dehazing. Most existing research on video dehazing is to speed up the process of dehazing. Sun et al. [17] proposed a real-time haze removal method based on bilateral filtering to reduce the processing time of 320 × 240 images to a speed of 20 frames per second. However, this method cannot satisfy the requirements of high-definition videos. Wang et al. [18] proposed a method based on Retinex theory that enhances image contrast in YUV color space and can process an image of 704 × 576 in 0.055 s. Kumari et al. [19] proposed an approach for dehazing images and videos based on a filtering method. The use of a gray-scale morphological operation made the approach faster, and it took only 80% of the execution time compared to a fast bilateral filter. Berman et al. [20,21] proposed a new method via calculating the air-light to dehaze fogs, which was based on a non-local prior. Their algorithm relies on the assumption that colors of a haze-free image are well approximated by a few hundred distinct colors that form tight clusters in RGB space. It performs well on a wide variety of images. However, these methods take every frame in videos as a single image, and they are completely based on image dehazing methods.

The characteristics of videos can be applied in specific video dehazing algorithms. Tarel et al. [22] proposed a video dehazing method for onboard video systems. This method can separate moving objects and driveway regions in videos and only update the depth information of moving objects. Zhang et al. [23] proposed a method based on spatial and temporal correlation that uses spatial and temporal similarity between frames to optimize the estimation of a scene depth map. Shin et al. [24] proposed an effective video dehazing technique to reduce flicker artifacts by using adaptive temporal average. However, these methods cannot remove the haze from videos in real time. Therefore, Kim et al. [25] proposed an image-dehazing method based on the image degradation model and kept a balance between image contrast enhancement and image information loss. To improve the speed of video dehazing, they adopted a video dehazing method by using temporal correlation, which can reach a speed of 30 frames per second for videos with a resolution of 640 × 480. However, this method adopts a fixed initial transmission value that cannot be adapted to images with different degrees of haze, and it cannot efficiently remove dense haze in videos. Our method uses an adaptive initial transmission value based on image characteristics to handle different degrees of hazes; meanwhile, it can reduce the processing time through lane space separation.

## 3. Single-Image Dehazing Using Adaptive Dark Channel Prior

### 3.1. Framework of Single-Image Dehazing Method

The most common dehazing model is based on atmospheric optics [26], which can describe the degradation process of a hazy image. In [27], the modeling function is simplified, and it is represented by Equation (1).

(1) I(p)=J(p)t(p)+A(1−t(p))

where p is a pixel in the image, I(p) and J(p) are the observed and haze-free image, respectively, A is the global atmospheric light, and t(p)∈[0,1] is the transmission map for each pixel that describes the proportion of the light arriving at a digital camera without scattering.

**Figure 1:**
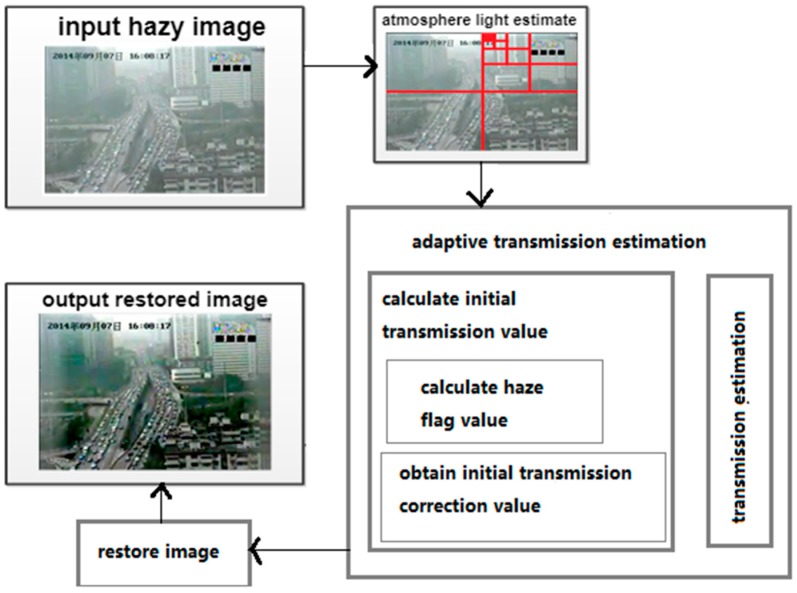
Framework of single-image dehazing method.

The process of haze removal for every frame of a traffic video can be divided into three steps: calculating atmospheric light, estimating the transmission map, and restoring the image. In this paper, we present a novel adaptive method for transmission map estimation, thus the dehazing algorithm can be applied to images with different degrees of haze. The framework of the single-image dehazing algorithm is shown in Figure 1.

We use a hierarchical searching method based on quad-tree subdivision [25] to find the areas least affected by haze and to get the brightest pixel in this area. The detailed steps are as follows:

Step 1: Divide an input image into four rectangular regions.
Step 2: Define the score of each region as the average pixel value subtracted from the standard deviation of the pixel values within the region.
Step 3: Select the region with the highest score and divide it further into four smaller regions.
Step 4: Repeat Steps 1 through Step 3 until the size of the selected region is smaller than a prespecified threshold. The prespecified threshold in this paper is 200, which is that the height * width of the selected region is smaller than 200.

At last, we choose the color vector, which minimizes the distance $\left|\left|\left(I_{r}\left(p\right),\;I_{g}\left(p\right),\;I_{b}\left(p\right)\right)-\left(255,\;255,\;255\right)\right|\right|$, where I(p) is the value of pixel p in the selected region as the atmospheric light.

### 3.2. Transmission Estimation for Enhancing the Contrast of Blocks

In general, a hazy block yields low contrast, and the contrast of a restored block increases as the value of the estimated transmission decreases. We adopt the image-contrast-enhanced method [18] to maximize the contrast of the restored blocks and get the best estimated transmission value.

Mean squared error contrast (CMSE) [28] can define the contrast of a restored block, which is represented by Equation (2):

(2) $$C_{MSE}=\overset{N}{\underset{p=1}{\sum }}{\frac{\left(J_{c}\left(p\right)-\bar{J_{c}}\right)}{N}}^{2}$$

where $J_{c}\left(p\right)$ represents the RGB color channel of pixel p in a block of input image, c∈{r,g,b}, $\bar{J_{c}}$ is the average value of $J_{c}\left(p\right)$, and N is the number of pixels in a block.

According to the assumption that the scene depths are locally similar [8,12,16], the dehazing algorithm in this paper determines a single transmission value for each block of size 32 × 32, and then gets the fixed optimal transmission value t for each block. For a pixel p in a block, t(p) in Equation (1) can be replaced with the fixed estimated transmission t of its block. Hence, $J_{c}\left(p\right)$ is represented by Equation (3).

(3) $$J_{c}\left(p\right)=\frac{I_{c}\left(p\right)-A}{t}+A$$

If Equation (3) is put into Equation (2), $C_{MSE}$ can be represented by Equation (4):

(4) $$C_{MSE}=\overset{N}{\underset{p=1}{\sum }}{\frac{\left(I_{c}\left(p\right)-\bar{I_{c}}\right)}{t^{2}N}}^{2}$$

where ${\bar{I}}_{c}$ is the average value of $I_{c}\left(p\right)$ in the input block. According to Equation (4), we can find that the mean squared error contrast is a decreasing function of t. Thus, we can select a small value of t to increase the contrast of a restored block. However, the value of t influences the pixel’s restored image value according to Equation (3).

However, when a block contains dense haze, it has a relatively narrow value range for input pixels. Thus, even though it is assigned a small t value, most of the input values are not truncated, and the block can be correctly restored. On the contrary, a block without haze usually has a broad range of values for input pixels and should be assigned a larger *t* value to reduce the information loss due to the truncation. Thus, we should not only enhance the contrast but also reduce the information loss.

Therefore, we need to set quantitative evaluations for contrast and information integrity. The contrast cost $E_{contrast}$ and the information loss cost $E_{loss}$ were proposed by Kim [25] to evaluate the contrast and information integrity, respectively.

(5) $$E_{contrast}=-\underset{c\in \left\{r,g,b\right\}}{\sum }\underset{p\in B}{\sum }\frac{\left(J_{c}\left(p\right)-\bar{J_{c}}\right)}{N_{B}}=-\underset{c\in \left\{r,g,b\right\}}{\sum }\underset{p\in B}{\sum }\frac{\left(I_{c}\left(p\right)-\bar{I_{c}}\right)}{N_{B}}$$

where ${\bar{J}}_{c}$ and ${\bar{I}}_{c}$ are the average values of $J_{c}\left(p\right)$ and $I_{c}\left(p\right)$ in block B, respectively, and $N_{B}$ is the number of pixels in B. Thus, we can maximize the mean squared error contrast by minimizing the value of $E_{contrast}$.

(6) $$E_{loss}=\underset{c\in \left\{r,g,b\right\}}{\sum }\underset{p\in B}{\sum }\{{\left(\min\{0,J_{c}\left(p\right)\}\right)}^{2}+{\left(\max\{0,J_{c}\left(p\right)-255\}\right)}^{2}\}$$

where $\min\{0,J_{c}\left(p\right)\}$ and $\max\left\{0,J_{c}\left(p\right)-255\right\}$ denote the truncated values for output pixels due to the underflow and overflow, respectively.

If we want to get a better restored image, the image contrast should be smoother, and the color information should be maintained as much as possible. Thus, these two factors should be taken into consideration synthetically, and the overall cost function is described as Equation (7).

(7) $$E=E_{contrast}+\lambda_{L}E_{loss}$$

where $\lambda_{L}$ is a weight coefficient that controls the relative importance of the contrast cost and the information loss cost [18]. The minimum value of E represents the most suitable contrast for restored images, and the color loss is as small as possible. Finally, for each block in a hazy image, we can get an optimal transmission $t^{*}$ by minimizing the value of E. The value of $t^{*}$ is the transmission we use while dehazing.

### 3.3. Adaptive Estimation of Initial Transmission

#### 3.3.1. Calculating Image Haziness Flag

We present a haziness flag T to measure the degree of haze in an image. The dark channel prior [9] can estimate the transmission of a block, which represents the luminosity of objects. The transmission has a close relationship with the degree of haze. Therefore, we can adopt the average value of transmission as the haziness flag *T* of an image. The haziness flag *T* is concerned with the effects of the degree of haze in images.

The dark channel prior is based on the observation that most local blocks in haze-free outdoor images contain some pixels that have very low intensities in at least one color channel. In other words, the dark channel value of a haze-free image is close to zero [9]. For any input image J, dark channel $J_{dark}$ can be expressed as Equation (8).

(8) $$J_{dark}\left(p\right)=\underset{y\in \Omega \left(p\right)}{min}\left(\underset{c\in \left\{r,g,b\right\}}{min}J_{c}\left(y\right)\right)$$

where c∈{r,g,b} and Ω(p) represent a local block centered at p, and y is a pixel in the local block Ω(p). A dark channel is the outcome of two minimum operators: $\underset{c}{min}J_{c}\left(y\right)$ is performed on each pixel, and $\underset{y\in \Omega \left(p\right)}{min}$ is a minimum filter [9].

Assuming that the atmospheric light $A_{c}$ is given, we can normalize the haze imaging Equation (1) by $A_{c}$ [9]:

(9) $$\frac{I_{c}\left(p\right)}{A_{c}}=t\left(p\right)\frac{J_{c}\left(p\right)}{A_{c}}+1-t\left(p\right)$$

Since the transmission t(p) is a constant $\tilde{t}\left(p\right)$ in local block, and the value of $A_{c}$ is given, the dark channel operation can be given by the following equations [9].

(10) $$\underset{y\in \Omega \left(p\right)}{min}\left(\underset{c}{min}\frac{I_{c}\left(y\right)}{A_{c}}\right)=\tilde{t}\left(p\right)\underset{y\in \Omega \left(p\right)}{min}\left(\underset{c}{min}\frac{J_{c}\left(y\right)}{A_{c}}\right)+1-\tilde{t}\left(p\right)$$

Using the concept of a dark channel [9], if $J_{c}$ is an outdoor haze-free image except for the sky region, the intensity of dark channel is low and tends to be zero, which leads to:

(11) $$\underset{y\in \Omega \left(p\right)}{min}\left(\underset{c}{min}\frac{J_{c}\left(y\right)}{A_{c}}\right)=0$$

Putting Equation (11) into Equation (9), we can eliminate the multiplicative term and estimate the transmission $\tilde{t}\left(p\right)$ simply by

(12) $$\tilde{t}\left(p\right)=1-\underset{y\in \Omega \left(p\right)}{min}\left(\underset{c}{min}\frac{I_{c}\left(y\right)}{A_{c}}\right)$$

where $\tilde{t}\left(p\right)$ is the predicted value of transmission of a block [9]. We need to calculate the average transmission for all blocks to obtain the average transmission T for the whole image, which is the value of image haziness flag.

#### 3.3.2. Correction of Initial Transmission

According to our experimental results, in a hazy image, the range of T is generally between 0.4 and 0.6. Although the image haziness flag T can characterize the nature of the image, taking T as the initial transmission value to get the optimal transmission leads to an excessive value of $t^{*}$. Thus, we set a correction value X, and set T∗X as the initial transmission value to decrease this initial value.

The structural similarity (SSIM) index is a method for predicting the perceived quality of digital television and cinematic pictures, as well as other kinds of digital images and videos. To guarantee that the restored images are closer to ground truths, we adopted the SSIM index [29] to measure the similarity between the ground truths and restored images. Because the traffic video is captured by a fixed camera, we can get a haze-free image of the same scene as a reference image in advance and compare the restored image with the reference image. The initial value of T can be obtained directly because it is relevant to the nature of images, whereas the unknown value X is calculated by the SSIM. In our experiments, we set X as a series of values between 0.3 and 1.2, and the interval is 0.02. Then, we take every X in this range multiplied by *T*, that is, T∗X, as the initial value of transmission and get the corresponding restored image. At last, we find a restored image that is closest to the haze-free image based on the maximum value of the SSIM index. Thus, the value of transmission is the optimal initial value, and the corresponding correction value X is the optimal correction value of initial transmission.

However, this method needs a haze-free image to get the optimal correction value X. This method is limited in practical applications, thus it is necessary to get the correction value according to the image characteristics. After analyzing the image contrast and the haze in images, we find the relationship between the correction value of initial transmission and the image characteristics. Therefore, a relatively reasonable initial transmission correction value can be obtained directly from hazy images.

If the relatively reasonable correction value of initial transmission is $X^{\prime }$, we take $T*X^{\prime }$ as the initial transmission value. Because the dehazing algorithm is based on the concept of enhancing the image contrast to the greatest degree, the contrast is the important indicator. The value of image haziness flag *T* represents the degree of haze that degrades the image contrast. Thus, the image contrast and haziness flag value should be considered simultaneously. We set C as the image contrast and set T∗C as a quantitative value representing the image characteristics. The constant value $X^{\prime }$ depends on the range of value T∗C.

**Figure 2:**
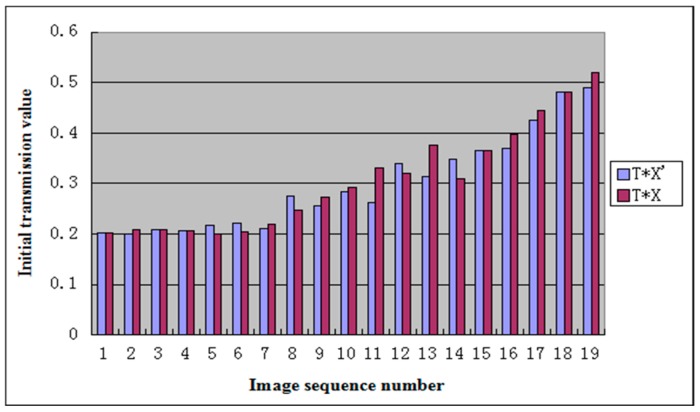
The histogram of *T* * *X* and *T* * *X*′.

Table 1 shows the values of $X^{\prime }$ for different ranges of T∗C. In Table 1, X is the optimal correction value obtained by the method with reference images, and $X^{\prime }$ is the relatively reasonable correction value obtained by the ranges of T∗C. In the dehazing algorithm, the initial transmission value is the key factor that affects the dehazing result. Table 1 shows the values of T∗X and $T*X^{\prime }$, which are the initial transmission value derived by optimal correction of initial X and relatively reasonable correction value $X^{\prime }$, respectively. Figure 2 shows the histogram of T∗X and $T*X^{\prime }$, where the values of T∗X and $T*X^{\prime }$ in the same group have similar values, and the difference of the values in the same group does not affect the dehazing results significantly. Therefore, our method can determine the optimal initial transmission value using only the nature of images and then obtain a more adaptive transmission value.

## 4. Adaptive Traffic Video Dehazing Method Using Spatial–Temporal Correlations

Compared with static traffic images, traffic videos have some unique characteristics. First, a traffic video is a collection of images with time continuity. Second, the cameras are fixed on the road and capture videos of the same scene over a long time, thus the videos are consistent in space. Therefore, we can use the correlations of spatial-temporal information to speed up traffic video dehazing.

### 4.1. Time Continuity of Traffic Videos

**Figure 3:**
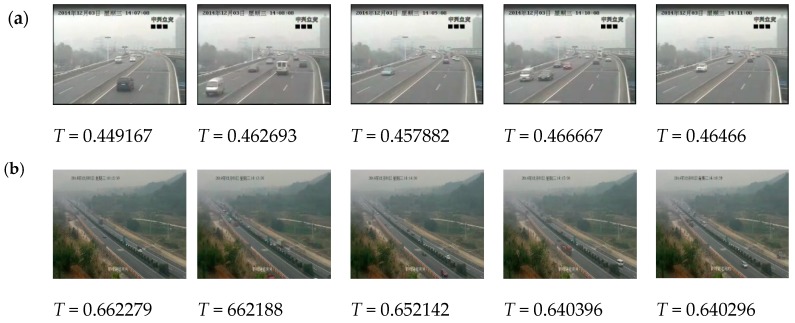
The difference of *T* for the images in a 5 min cycle. The images come from different scenes (**a**,**b**).

**Figure 4:**
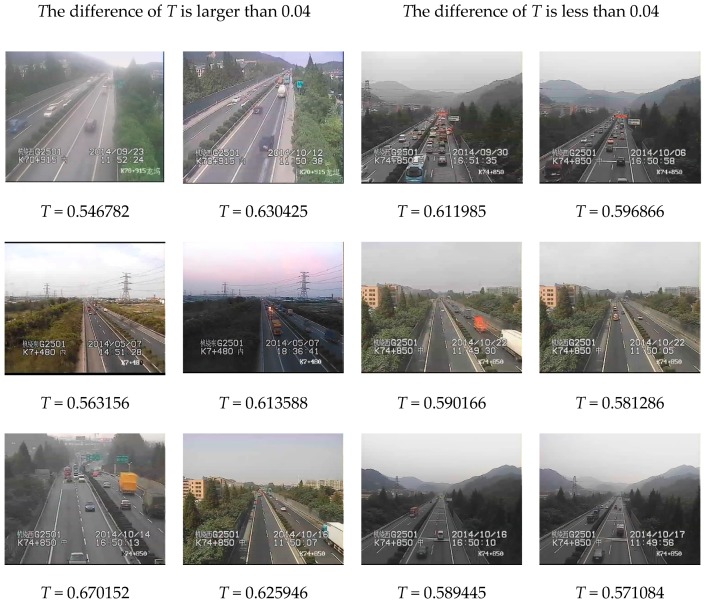
The images with different *T* values.

Because the cameras are fixed, the scenes in traffic videos barely change over a long period of time, and the influence of haze is stable. In our experiments, we use the traffic videos from ZhongHe elevated freeways in Hangzhou City, set a cycle of five minutes, and regard the frames in one cycle as a collection of images with the same characteristics. Figure 3 shows images whose interval is 1 min in a 5 min cycle, and the difference of T is very small, usually less than 0.04. Figure 4 presents the difference in restored images by using different T values where the results have no obvious influence on visibility with the difference of T less than 0.04. Therefore, if the videos are captured at the same scene, the values of T for these video images in a 5 min cycle are at the same level, and the cycle of 5 min is reasonable in practical application.

After setting the 5 min cycle, we can take the first frame of a video segment as a reference frame. We can determine the image haziness flag value T and the relatively reasonable initial transmission correction value $X^{\prime }$ from the reference frame and then calculate the optimal transmission $t^{*}$. In this way, we can speed up the dehazing processing for the traffic video. This method can avoid incorrect transmission estimation, which is caused by the changes in atmospheric light, and eliminate the discontinuity of videos after dehazing.

### 4.2. Transmission Refinement Based on Spatial Structure

We estimate the optimal transmission based on the assumption that all pixels in a block have the same transmission. However, scene depths may vary spatially within a block, and the block-based transmission map usually has a blocking-artifact problem. Therefore, an edge-preserving filter is adopted to refine the block-based transmission map.

The single-image dehazing method using dark channel prior [9] employs the soft matting technique [30] to refine the large block size in the transmission map, which causes an enormous computational burden. In this paper, the guided filter method [31] is adopted to refine the transmission map, which has less computational cost. The filtered transmission $\hat{t}\left(p\right)$ is an affine combination of the guidance image I(p), as show in Equation (13):

(13) $$\hat{t}\left(p\right)=s^{T}I\left(p\right)+\psi$$

where $s={\left(s_{r},s_{g},s_{b}\right)}^{T}$ is a scaling vector, and ψ is an offset determined by the size of block. For a block in one image, the optimal parameters of $s^{*}$ and $\psi^{*}$ can be obtained by minimizing the difference between the transmission t(p) and the filtered transmission $\hat{t}\left(p\right)$ using the least squares method as Equation (14):

(14) $$\left(s^{*},\psi^{*}\right)=argmin(s,\psi )\underset{p\in \Omega }{\sum }{\left(t\left(p\right)-\hat{t}\left(p\right)\right)}^{2}$$

If the transmission is too small, the noise will be enhanced in the restored image [9]. Thus, the lower limit of the transmission is set to 0.1. If a window slides pixel by pixel over the entire image, there will be multiple windows that overlap at each pixel position. Therefore, we adopt the centered window scheme, which sets the final transmission values as the average of all associated refined transmission values at each pixel position. However, the average transmission value in this scheme will cause blurring in the final transmission map, especially around object boundaries, where the depths change abruptly. To overcome this problem, the shiftable window scheme [32] is employed instead of the centered window scheme. The centered window scheme overlays a window on each pixel so that the window contains multiple objects with different depths, which leads to unreliable depth estimation. In the shiftable window scheme, the window is shifted within a block of 40 × 40. The optimal shift position is selected depending on the smallest change of pixel values within the window. Even though a shiftable window is selected for a specific pixel, the number of overlapping windows usually varies at different positions. The windows in smooth regions are selected more frequently than those in rough boundary regions. Thus, the shiftable window scheme can reduce the effects of unreliable transmission values derived from rough boundary regions, thereby alleviating the blurring artifacts.

### 4.3. Lane Separation for Traffic Videos

After analyzing the spatial characteristics of traffic video, we found that the traffic lane is an obvious structure. In a traffic video detection system, the detected objects are mostly concentrated in the driveway regions. The areas outside lanes are not the regions of interest in traffic video processing. Therefore, we can process haze removal only in the driveway region of traffic video to reduce computing time.

However, the estimations of atmospheric light and transmission are based on the whole image. If these values are achieved only through the driveway regions, it may cause some deviations, especially when the sky occupies a large area of the image, such as the cases shown in Table 2. The larger the sky region is, the greater the deviation for the value of T∗X is. Therefore, the separated lane can be used in the last step to restore the pixels only for the driveway regions.

**Figure 5:**
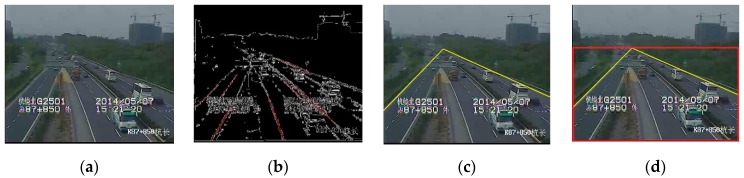
Lane space separation: (**a**) original Image; (**b**) lane candidates; (**c**) driveway boundary; (**d**) result for lane separation.

We adopt a straight-line extraction algorithm based on the Hough transform to detect the lanes and separate the driveway region from the global image. The process of haze removal combined with the driveway region separation is described as follows:

1. Calculate the global atmospheric light A, the value of haziness flag T, and the image contrast C, then estimate the optimal transmission map for each block in an image.
2. Get the driveway region, as shown in Figure 5.
  Step 1: Obtain the edge information in the video through edge detection.
  Step 2: Remove obviously wrong-angle lines by Hough linear fitting, and obtain lane candidates, as shown in Figure 5b.
  Step 3: Find the far left lane and the far right lane, and set them as the driveway boundaries, then find the intersection of these two lines, as shown in Figure 5c.
  Step 4: Identify a rectangular area as the driveway region, which is composed of the boundary of the image and a horizontal line across the intersection, as shown in Figure 5c. If the intersection is outside the image, take the whole image area as the driveway region.
3. Use the original pixel values and the optimal transmission of driveway region in the dehazing model to restore the image in the driveway region.

**Figure 6:**
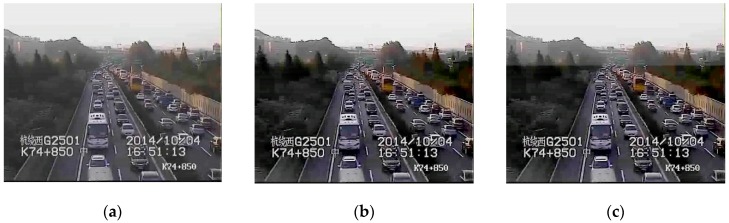
Results for video dehazing with lane separation: (**a**) before haze removal; (**b**) haze removal without lane separation; (**c**) haze removal with lane separation.

In a traffic video detection system, each camera is located at a fixed position and captures the same traffic scenes for a long time. Based on the time continuity, the result of lane space separation for the initial frame of a traffic video can be used over a long time period. Lane space separation can decrease the area of haze removal and improve the efficiency of the dehazing algorithm. Figure 6 shows the haze removal results with and without lane separation. In this scene, the dehazing of 2000 frames needs 35.301 s without lane separation and 32.74 s with lane separation (lane space separation takes 0.182 s). Although lane separation requires some time, the operation just occurs in the first frame. Thus, the time for lane separation can be shared by all frames of a traffic video. With an increasing number of frames, the efficiency of the dehazing algorithm with lane separation will be improved more significantly. Hence, if the driveway region is a larger portion of a whole image, the processing time can be decreased obviously. When real-time processing is required, a little reduction in processing time has been of practical significance.

### 4.4. Optimization Based on Spatial Distribution of Cameras

With an increasingly complex layout of transportation networks, the number of traffic monitoring cameras also increases gradually, and sometimes there are multiple cameras in the same section of road. These cameras located in close physical proximity usually have the same hardware indicators. In a traffic video detection system, multiple cameras are connected to one system. These cameras have similar characteristics according to their spatial distribution. The weather is also an index with spatial characteristics, that is, the degrees of haze are similar in nearby regions. Thus, we can use the spatial distribution information of cameras to speed up dehazing and optimize the performance of the traffic video detection system.

**Figure 7:**
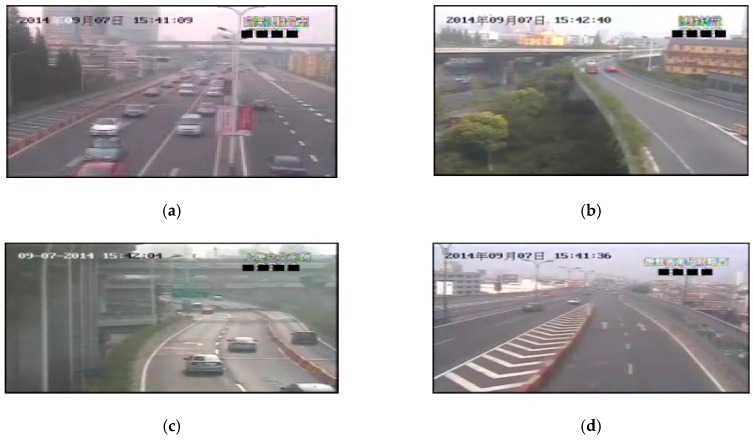
Example images of the nearby regions.

**Figure 8:**
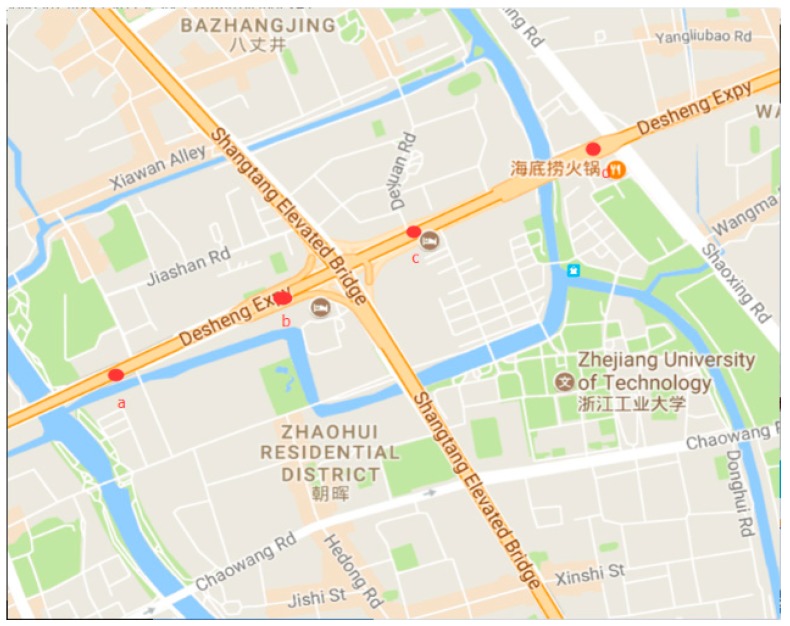
The locations of cameras.

Figure 7 shows the images captured by four surveillance videos of DE-elevated freeways in Hangzhou City at the same time. The locations of these cameras are shown in Figure 8, where the distance between the cameras is about 500 to 600 m. Table 3 shows the initial transmission values of these four videos. The haziness flag values *T* calculated from each video are shown in the first column of Table 3. We obtain relatively proper initial transmission correction value $X^{\prime }$ by using the method proposed in Section 3, and then determine the initial transmission value T∗X. According to the results, these initial transmission values are very numerically similar, thus there may be no obvious influence on the restored images.

**Figure 9:**
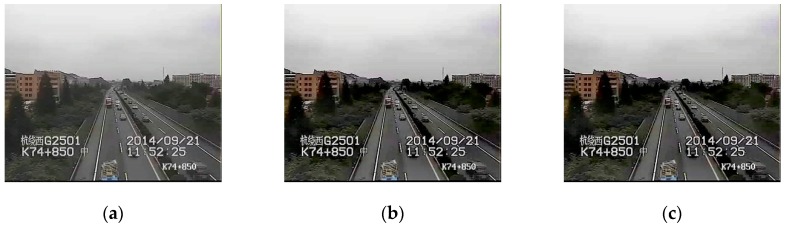
Results of haze removal with and without calibration camera: (**a**) original image; (**b**) initial transmission value for calibration camera is 0.596; (**c**) initial transmission value for image itself is 0.578.

In traffic video dehazing, the cameras are divided into different regions according to their locations, and one camera in a region is set as the calibration camera. The images from the calibration camera are used to calculate the initial transmission value, which is also applied to other cameras in the same region. Therefore, we can avoid repeatedly calculating the values of T, C, and $X^{\prime }$ for other cameras, thus improving the efficiency of haze removal. The results of haze removal with the initial transmission value obtained by calibration cameras is shown in Figure 9b, and the result directly using the initial transmission value obtained by the image itself is shown in Figure 9c. It is obvious that the results are very similar in these two ways. It takes 0.033 s to calculate the initial transmission value, which can be saved by using that of the calibration camera.

## 5. Results

In the efficient traffic video dehazing method using adaptive dark channel prior and spatial-temporal correlations, a video sequence is converted into YUV color space where Y represents the luminance and U/V represents the chromaticity. Human eyes are more sensitive to high-frequency signals than low-frequency signals and more sensitive to changes in visibility than changes in color. The U and V components are less affected by haze than the Y component. Thus, we can only adopt the luminance (Y) component to reduce computational complexity. In our experiments, we implemented each method with Opencv and C/C++ language. The source codes were compiled with Microsoft Visual Studio 2010 and run on an Intel Core I5-2400 processor and 4 GB of main memory running a Windows 7 system.

### 5.1. Results for Single Image Dehazing

**Figure 10:**
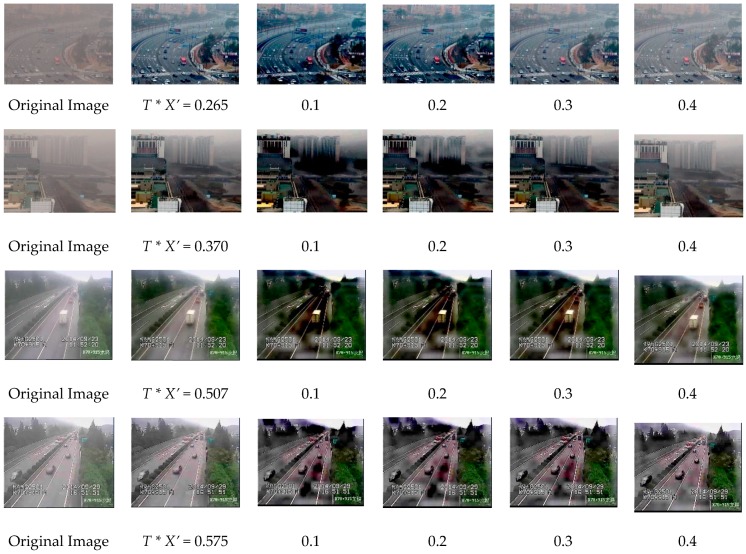
Results for different initial transmission using our adaptive method.

Our adaptive method can determine the initial transmission according to the image characteristics, thus it can produce a more satisfactory dehazing result than the method with fixed initial transmission. Figure 10 shows the restored images using our adaptive method, and there are four different initial transmission values, 0.1, 0.2, 0.3, and 0.4. It is obvious from the experimental results that the smaller initial transmission values may lead to some blocks in the images with overstretched contrast, therefore the optimal initial transmission for the first image is between 0.2 and 0.3, the value for the second image is between 0.3 and 0.4, and the value for the third and fourth images is above 0.4. The $T*X^{\prime }$ values for the images obtained by our method are all located in the range of the optimal initial transmission. Therefore, our method is adaptable for images with different degrees of haze.

**Figure 11:**
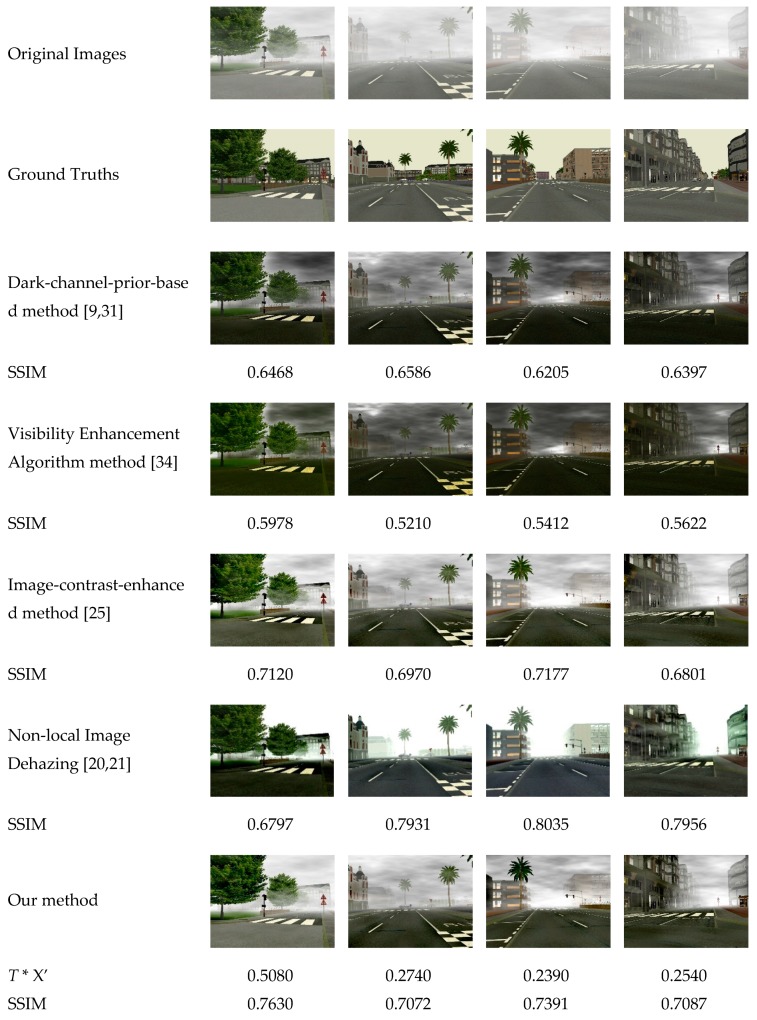
Comparison of the restored images using different methods; * SSIM = structural similarity.

Figure 11 shows four images from Foggy Road Image Database (FRIDA) [33] and restored these images using the dark-channel-prior-based method [9,31], the visibility enhancement algorithm [34], the image-contrast-enhanced method [25], the non-local image dehazing method [20,21], and our method. The SSIM values in Figure 7 are the average values of three channels of RGB. In FRIDA [33], each image without fog is associated with some hazy images, and different kinds of fog are added in each image—uniform fog, heterogeneous fog, cloudy fog, and cloudy heterogeneous fog. According to the experimental results, the dark-channel-prior-based method does not have satisfactory results for haze removal in heterogeneous fog and cloudy heterogeneous fog, while the image-contrast-enhanced method and our method achieves more satisfactory results for these two cases. In addition, our method obtains the highest SSIM for the restored images compared to the first three methods, thus the restored images using our method are more similar to ground truth. As to the results of non-local image dehazing method [20,21], the SSIM for some restored images may be higher than those of our method. However, the non-local image dehazing method takes longer processing time, as shown in Table 4. Table 4 provides the overall processing times of these methods. Our method is faster than the dark-channel-prior-based method [9,31] and visibility enhancement algorithm [34]. However, our method takes more time than the image-contrast-enhanced method [25] because it spends some time in calculating the image haziness flag value and the initial transmission correction value. However, the results for haze removal using the proposed method are better than the results of the image-contrast-enhanced method. Although the non-local image dehazing method can get more satisfactory restored images, it is too slow to be used in real-time scenarios. In addition, it usually needs to manually set the parameters to different scenes, which is not suitable for real-time traffic video processing. Further still, we can spread this part of the computation time over all frames in video dehazing and reach a faster dehazing speed through the fusion of spatial and temporal information.

### 5.2. Results for Traffic Video Dehazing

**Figure 12:**
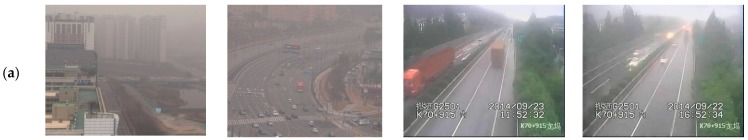

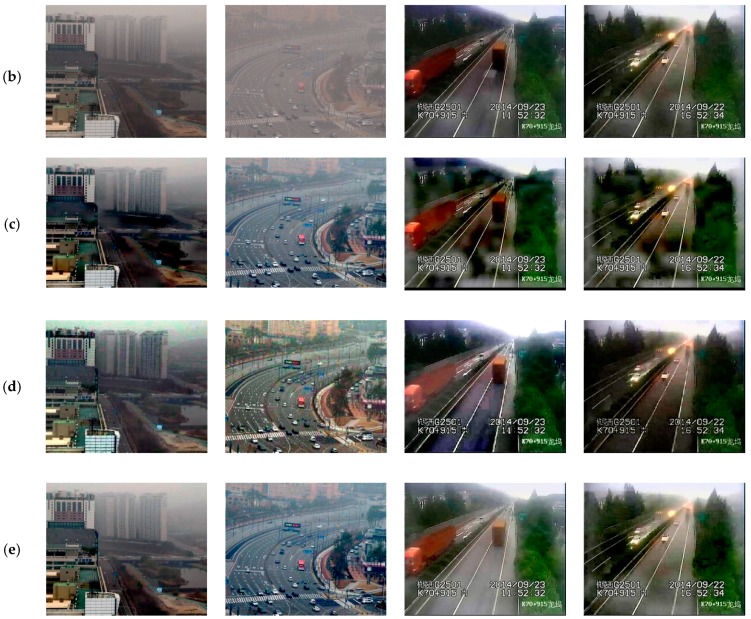
Comparison of restored videos. (**a**) Original Videos; (**b**) Dark-channel-prior-based method; (**c**) Image-contrast-enhanced method; (**d**) Non-local Image Dehazing; (**e**) Our method.

To get better restored images, we restore the whole image for the first frame of a time slice and use the area outside the lane space of the restored frame to replace those areas of the following frames. Moreover, we adopt the parallel programming tools SIMD [35] and OpenMP [36] for rapid calculation. Figure 12 presents a comparison of three approaches for traffic video dehazing, where Figure 12a shows the original videos; Figure 12b shows the results for the dark-channel-prior-based method with guided filtering [9,31], which uses the transmission map obtained from the first frame to filter the following frames; Figure 12c shows the results for the image-contrast-enhanced method [25], whose initial transmission is a constant value 0.3; Figure 12d shows the results produced by our method. Experimental results demonstrate that the image-contrast-enhanced method leads to some blocks with overstretched contrast, such as the images in groups (1), (3), and (4). For some urban scenes, the color is not obviously different between the driveway and background, such as the examples in group (1) with medium haze and group (2) with dense haze. Our method can restore these videos in a manner more similar to the haze-free scenes, and the driveway and the vehicles can been seen more clearly. However, the dark-channel-prior-based method cannot deal with these videos. For the suburban scenes where the trees and road surface are obviously different in color, such as images in group (3) that were captured in daytime and images in group (4) that were captured in dense haze with vehicle headlights on, our method achieves better restored results than the other two methods. For the restored images using our method in group (3), the driveway color is more uniform. For the restored images using our method in group (4), there are no blocks with overstretched contrast, and the color of trees with hierarchical structure is more realistic. Therefore, our method can maintain the image details and restore images that are more similar to the real scene with proper contrast.

As we can see from the experiment results, our method produces better haze removal results by determining parameters according to image characteristics. It is also applicable to dense fog or a variety of fog densities. Moreover, it makes the restored images more similar to the real scene and avoids the problem that the restored images exhibit overstretched contrast. Therefore, it can solve the general problems in the existing dehazing algorithms—contrast distortion after video dehazing and failure to remove dense haze.

In addition, our method adopts the spatial correlation, time continuity, lane separation, and spatial distribution of cameras to improve computational efficiency. Besides the processing time, the performance parameters of frames per second (fps) and SSIM of different methods for the video dehazing in Figure 12 are shown in Table 5. In order to meet the actual traffic scenarios, we process the video frame by frame, and the data show the total processing time for 1000 frames. Our method uses the initial frame in a time slice to calculate the transmission map and atmospheric light and adopts the lane separation to decrease the dehazing areas. Compared with other methods, the time of dehazing in our method decreases when the time slice increases. According to the experiment results, our method can obviously speed up video dehazing, especially if the video has high resolution or the driveway is only a small part of the whole image. Our method can restore the video with a resolution of 720 × 592 at about 57 fps, nearly four times faster than dark-channel-prior-based method and one time faster than image-contrast-enhanced method. Furthermore, our method obtains the highest SSIM for the restored videos compared with other existing methods, thus the restored videos using our method are more similar to ground truth. Therefore, the proposed method not only has superior haze removing and color balancing capabilities but also restores and enhances the degraded videos in real time.

## 6. Conclusions

Traditional haze removal methods fail to restore the images with different degrees of haziness in a real-time and adaptive manner under most circumstances. To solve this problem, we propose an efficient traffic video dehazing method using adaptive dark channel prior and spatial-temporal correlations. The dark channel prior is based on the statistics of outdoor haze-free images, but it cannot adaptively estimate the initial transmission value based on the degree of haze and contrast of images. Therefore, we adopt the image-contrast-enhanced method to obtain the best estimated transmission value as the initial transmission value of dark channel prior. The image dehazing method using adaptive dark channel prior can overcome the shortcomings of existing dehazing algorithms that overstretch contrast after haze removal and deal with images with dense haze to a satisfactory level. Additionally, we introduce the temporal-spatial correlation of traffic videos to speed up the traffic video dehazing using the time continuity to set a time slice, the characteristics of block structure to refine transmission, lane space structure to decrease the restored area, and multi-camera distribution to simplify the calculation of parameters. The experiment results show that our method can restore satisfactory image appearance, which can remove dense haze effectively and does not produce results with overstretched contrast. The temporal and spatial characteristics can reduce the computation time, especially for dehazing multiple videos.

However, the dark channel prior is a kind of statistic, and it may not work for some particular traffic videos. When there are rapidly changing hazes in the videos, the dark channel of the scene radiance has a great difference at different times. In addition, if the scene objects are inherently similar to the atmospheric light and no shadow is cast on them, the adaptive dark channel prior is invalid. The dark channel of the scene radiance has bright values near such objects. As a result, our method may underestimate the transmission of these objects and overestimate the haze layer.

## Figures and Tables

**Table 1:** The value of *x’* for different ranges of T * C.

Image No.	*T*	*C*	T * C	*X*	The Range of T * C	*X*′	*T* * *X*′	*T* * *X*
1	0.4032	3.8224	1.5414	0.50	T * C < 10	0.5	0.2016	0.2016
2	0.4006	6.3436	2.5410	0.52		0.5	0.2003	0.2083
3	0.4177	8.4845	3.5437	0.50		0.5	0.2088	0.2088
4	0.4113	13.4080	5.5151	0.50		0.5	0.2056	0.2057
5	0.4329	13.2774	5.7476	0.46		0.5	0.2164	0.1991
6	0.4444	17.6432	7.84004	0.46		0.5	0.2222	0.2044
7	0.4211	19.7160	8.3039	0.52		0.5	0.2160	0.2190
8	0.4584	22.1363	10.1480	0.54	10 ≤ T * C < 15	0.6	0.2750	0.2476
9	0.4275	25.5289	10.9141	0.64		0.6	0.2565	0.2736
10	0.4732	26.9131	12.7346	0.62		0.6	0.2839	0.2934
11	0.4370	31.9037	13.9419	0.76		0.6	0.2622	0.3321
12	0.4862	31.3389	15.2359	0.66	15 ≤ T * C < 20	0.7	0.3403	0.3209
13	0.4469	38.3871	17.1555	0.84		0.7	0.3128	0.3754
14	0.4987	35.6754	17.7904	0.62		0.7	0.3491	0.3092
15	0.4555	44.9152	20.4609	0.80	20 ≤ T * C < 25	0.8	0.3644	0.3644
16	0.4625	50.9075	23.5422	0.86		0.8	0.3700	0.3977
17	0.4724	57.3643	27.1012	0.94	25 ≤ T * C < 30	0.9	0.4252	0.4441
18	0.4812	63.6731	30.6395	1.00	T * C ≥ 30	1.0	0.4812	0.4812
19	0.4909	70.3751	34.5454	1.06		1.0	0.4909	0.5203

**Table 2:** Global image and driveway.

Regions	Parameters	Case 1	Case 2	Case 3	Case 4
		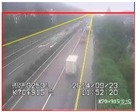	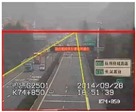	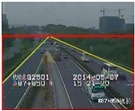	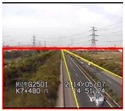
Driveway Region	*T*	0.590856	0.704105	0.839763	0.83898
	contrast	47.7547	49.0273	54.0312	62.208
	*X*′	0.90000	1.0000	1.0000	1.0000
	*T* * *X*’	0.53200	0.70400	0.8400	0.8390
Global Image	*T*	0.563265	0.632323	0.773405	0.563549
	contrast	48.8811	49.2619	57.5056	127.7800
	*X*’	0.9000	1.0000	1.0000	1.0000
	*T* * *X*’	0.5070	0.6320	0.7730	0.5660

**Table 3:** Initial transmission values for videos in nearby regions.

Cases	Haze Flag Value *T*	Initial Transmission Correction Value *X*′	Initial Transmission Value *T* * *X*′
a	0.524188	1	0.524
c	0.580732	1	0.581
b	0.569918	1	0.570
d	0.517431	1	0.517

**Table 4:** Processing times for single-image dehazing.

Image Resolution	Dark-Channel-Prior Method [9,31]	Visibility Enhancement Algorithm [34]	Image-Contrast-Enhanced Method [25]	Dehazing Only Using Adaptive Dark Channel Prior	Non-Local Image Dehazing [20,21]	Our Method
640 × 480	0.897 s	1.014 s	0.396 s	0.506 s	2.546 s	0.433 s
480 × 400	0.516 s	0.895 s	0.165 s	0.301 s	2.387 s	0.252 s
320 × 240	0.173 s	0.348 s	0.057 s	0.262 s	2.024 s	0.211 s

**Table 5:** Comparing the performance parameters.

Case	Image Resolution	He et al. [9,31]			Kim et al. [25]			Our Method		
		Time	fps	SSIM	Time	fps	SSIM	Time	fps	SSIM
(1)	640 × 480	66.787s	15.0	0.6870	35.359 s	28.3	0.6990	17.507 s	57.1	0.7012
(2)	640 × 480	64.576 s	15.4	0.7002	34.471 s	29.0	0.7079	18.005 s	55.5	0.7232
(3)	720 × 592	95.638 s	10.5	0.6155	37.858 s	26.4	0.6322	17.604 s	56.8	0.6488
(4)	720 × 592	90.911 s	11.0	0.5932	39.855 s	25.1	0.6011	16.925 s	59.0	0.6155
